# Psychological Distress and Coping Among Dental Practitioners During the COVID-19 Pandemic: A Survey From India

**DOI:** 10.7759/cureus.28023

**Published:** 2022-08-15

**Authors:** Mahendra Sahu, Ajay Kumar, Santhosh Rao, Purushotham A

**Affiliations:** 1 Dentistry, All India Institute of Medical Sciences, Raipur, IND; 2 Psychiatry, All India Institute of Medical Sciences, Raipur, IND

**Keywords:** coping skills, dental practice, dental procedures, psychological issues due to covid-19, sars-cov-2 pandemic

## Abstract

Aim: Most dental procedures are aerosol-generating and hence highly risky for spreading SARS-CoV-2 (COVID-19) infection. This can lead to sufficient psychological distress, avoidance of risky procedures, and impact on dental practice. We intend to examine the effect of the COVID-19 pandemic on dental practice and psychological distress among dental practitioners.

Methods: An online survey was conducted by an email-based survey link; 1257 registered dental practitioners were contacted across the country.

Results: Most dental practitioners continue to practice during the COVID-19 pandemic (81.08%). Postgraduate specialists significantly outnumber undergraduates in performing dental procedures (p=.001). Career-related anxiety was considerably high among postgraduates (61.96%; p=.036) during the initial phase of the SARS-CoV-2 pandemic in India. In contrast, self-efficacy was significantly better among postgraduates than undergraduates (p=.027).

Conclusion: Dental practitioners suffered considerable impact due to the COVID-19 pandemic. It is important to enhance coping and self-efficacy strategies among dental practitioners.

## Introduction

The World Health Organization (WHO) officially declared the SARS-CoV-2 (COVID-19) outbreak a pandemic on March 11, 2020. Several preventive measures and restrictions were imposed to prevent the spread of the SARS-CoV-2 (COVID-19) virus. In India, a nationwide lockdown was declared on March 24, 2020 for 21 days [1-3]. The devastating effect of the COVID-19 pandemic was seen across all strata of lives, and the threat was palpable to all categories of health professionals and medical specialties [4]. 

The COVID-19 pandemic significantly affected dental practice; dental practitioners suffered a great amount of COVID-19-related anxiety and fear since the COVID-19 virus primarily spreads through aerosol, and most dental procedures are aerosol generating [5,6]. It was observed that most dental procedures were either postponed or limited to oral surgeries only during the COVID-19 pandemic [7-9]. Several safety guidelines were released to ensure that dental practitioners continued dental care services safely [7].

There are around 117,825 registered dental surgeons in the country; it is pertinent to assess the pattern of dental practices and psychological distress related to dental procedures during the COVID-19 pandemic [10]. We hypothesized that dental practice sustained a significant impact, and dental practitioners were subjected to substantial psychological distress during the COVID-19 pandemic. To validate our hypothesis, we conducted an online survey from August-September 2020, i.e. at the peak of India's first wave of the COVID-19 pandemic.

## Materials and methods

Subject and participants

The institutional ethics committee of the All India Institute of Medical Sciences, Raipur approved the study (AIIMSRPR/IEC/2020/597). The study was conducted using - the SurveyMonkey® platform. Participants who were undergraduates or above in dentistry, registered with the Dental Council of India, and currently practicing in India were invited by email. A WhatsApp® invitation was sent to those whose email addresses could not be found. Participants who did not practice dentistry (due to any reason other than the COVID-19 pandemic), did not provide consent or did not complete the survey were excluded. The invitation link and IP address were anchored to the collector link to avoid duplicate responses. A weekend reminder was sent to only those who did not respond to the first invitation. The invitation link remained open from August 27, 2020, 5:00 PM IST to September 15, 2020, 10 AM IST (18 days and 17 hours). After clicking on the invitation link, the participants were directed to the invitation page; the invitation page provided detailed information about the study, followed by the participant's consent.

Survey/questionnaire

A questionnaire composed of 32 items was used to collect the sociodemographic details (10 items), factors related to the COVID-19 pandemic, and fear and anxiety associated with COVID-19 (12 items). The questionnaire was distributed to 10 doctors of the same institution, their opinion and suggestions were collected separately, and finally, the questionnaire was used. The self-efficacy scale, a 10 items scale with 1 to 4 scores for each item, is used to assess the coping skills of the dental practitioners. A higher score on the self-efficacy scale indicates lower psychological distress and better general coping skills [11,12]. The average time taken by the participants to complete the survey was six minutes.

Statistical analysis

Continuous variables were reported as means with standard deviation (SD), and categorical variables were reported as a number with the percentage of the total. Chi‑square test was used to assess the statistical significance of the distribution pattern of various variables. The analysis was carried out using Statistical Package for the Social Sciences, version 20.0 (SPSS, IBM Corp., Armonk, NY).

## Results

A total of 1257 registered dental practitioners were invited to participate by disseminating invitation links through email and weblink in WhatsApp® messages; a total of 742 responses, 228 by email, and 514 weblinks, with a response rate of 71% was received. Finally, 518 participants completed the survey and their responses were included in the final analysis.

The mean age of the participants was 33.69 ± 8.95 years; 50.19% were males (50.19%) and 56.76% were married. The graduates of Master of Dental Surgery (MDS) outnumbered (81.08%) the graduates of Bachelor of Dental Surgery (BDS) (32.04%). Most participants were involved in private practice (44.21%) and did not have risk factors like age of more than 60 years, uncontrolled diabetes, hypertension, or current treatment with immunosuppressant drugs (83.88%) (Table 1).

**Table 1:** Sociodemographic details of the participants (n=518) SD: Standard Deviation

Variables		Frequency n (%) / mean (± SD)
Age (years)		33.69 (±8.95)
Gender	Male	260 (50.19%)
	Female	258(49.80%)
Relationship status	Single	220(42.47%)
	Married	294(56.76%)
	Separated	3(0.58%)
	Widow	1(0.19%)
Degree/Speciality	Conservative Dentistry & Endodontics	52 (10.04%)
	Prosthodontics	37 (7.14%)
	Orthodontics	51 (9.85%)
	Oral & Maxillofacial Surgery	119 (22.97%)
	Pedodontics	32 (6.18%)
	Periodontics	18 (3.47%)
	Oral Medicine and Radiology	12 (2.32%)
	Community Dentistry	20 (3.89%)
	Oral Pathology	11 (2.12%)
	Bachelor of Dental Surgery (BDS)	166 (32.05%)
Occupation	Private practitioner	229 (44.21%)
	Government employee	137 (26.45%)
	Private dental college employee	148 (28.57%)
	Retired	4 (0.77%)

Most practitioners continued their practice (81.08%) with a significantly higher proportion of males and with postgraduate qualifications (χ²=37.21, p=<.001 and χ²= 10.68, p=.001) during the COVID-19 pandemic, performing all sorts of dental procedures while limiting themselves to the non-aerosol generating procedure (Table 2).

**Table 2:** Impact of COVID-19 pandemic on dental practice **P<0.05, **P<0.01, ***P<0.001

S. No.	Questions	Frequency "yes" n(%)	Chi-square test/t-test (p)
1.	Are you practising during the COVID19 pandemic?	420 (81.08)	
	Male	238 (45.94)	37.21 (.001)
	Female	182(35.13)	
	Postgraduates	299 (57.72)	10.68(.0010)*
	Undergraduates	121(23.33)	
2.	Are you performing all sorts of oral procedures during COVID 19 Pandemic?		
	Male	147(28.37)	6.064 (.013)*
	Female	173(33.39)	
	Postgraduates	159(30.69)	1.3449(.246)
	Undergraduates	66(12.74)	
3.	Are you limiting your dental practice only to non-aerosol generating procedures?		
	Male	147 (28.37)	6.064(.013)*
	Female	173 (33.39)	
	Postgraduates	212(40.92)	1.115(.290)
	Undergraduates	108(20.84)	
4.	Do you have any co-morbid conditions like age above 60 years, uncontrolled diabetes, hypertension, immunosuppressant therapy or any other conditions that are classified as a high-risk group?		
	Male	45(8.68)	0.4577(.49)
	Female	39(7.52)	
	Postgraduates	58 (11.19)	0.055(.81)
	Undergraduates	26 (5.01)	
5.	Do you have any child of age 5 years or less in your family?		
	Male	96(18.53)	7.75(.0053)**
	Female	65 (12.54)	
	Postgraduates	106(20.46)	0.688(.406)
	Undergraduates	56 (12.54)	
6.	Do you have any family members of age 50 years or more in your family?		
	Male	234 (45.17)	.355(.550)
	Female	228(44.01)	
	Postgraduates	315 (60.81)	0.102 (.749)
	Undergraduates	147 (28.37)	

As much as 61.97% expressed concern about getting COVID-19 infection while 50% were concerned about losing their job or shut-down of a privately owned clinic as well as anxiety related to their career. We found a significant difference in career-related anxiety levels between postgraduates and undergraduates (χ² =8.49, P=.036) (Table 3).

**Table 3:** COVID-19-related anxiety among postgraduates was 352 (67.95%) and undergraduates (BDS) at 166 (32.04%) **P<0.05, **P<0.01, ***P<0.001 BDS: Bachelor of Dental Surgery

S No.	Variable	Not at all n(%)	Normal n(%)	Much n(%)	Very much n(%)	Chi-square test/t-test (p)
1.	Are you anxious to be infected with COVID-19 because of your profession?	42(8.11)	155(29.92)	158(30.50)	163(31.47)	
	Postgraduates	26(61.90)	105(67.74)	113(71.51)	108(66.26)	1.84(.604)
	Undergraduate: BDS	16(38.10)	50(32.26)	45(28.49)	55(33.74)	
2.	Are you anxious about shutting your clinic or loosening your job because of the COVID-19 outbreak?	110(21.24)	154(29.73)	133(25.68)	121(23.35)	
	Postgraduates	74(67.28)	115(74.68)	84(63.16)	79(65.29)	5.016(.1705)
	Undergraduate: BDS	36(32.72)	39(25.32)	49(36.84)	42(34.71)	
3	Are you anxious about your career as a dentist because of the COVID-19 outbreak?	103(19.88)	135(26.06)	127(24.52)	153(29.54)	
	Postgraduates	75(72.82)	100(74.07)	86(67.72)	91(59.48)	8.49(.036)*
	Undergraduate: BDS	28(27.18)	35(25.93)	41(32.28)	62(40.52)	

To manage cases of COVID-19, sufficient personnel protective equipment was present in their clinical set-up. Female dental practitioners expressed higher career-related anxiety compared to their male counterparts, as shown by their response to the question - "Are you anxious about your career as a dentist because of the COVID-19 outbreak?" (χ² =9.26, p=.026) (Table 4). 

**Table 4:** COVID-19-related anxiety across genders **P<0.05, **P<0.01, ***P<0.001

S. No	Variables	Male frequency (260) n(%)	Female frequency (258) n(%)	Chi-square test/t-test (p)
1.	Do you feel that you have an adequate PPE kit, N95 mask, face shield, goggle, and fumigator in your hospital or clinic for managing COVID19 patients?			
	Not at all	34(13.08)	42(16.34)	18.39(.001)
	Normal	92(35.38)	127(49.22)	
	Much	67(25.77)	55(21.31)	
	Very much	67(25.77)	34(13.17)	
2.	Do you feel that you have sufficient knowledge for managing COVID19 patients in your setup?			
	Not at all	11(4.23)	24(9.30)	36.54(.001)
	Normal	95(36.54)	147(56.91)	
	Much	111(42.69)	70(27.13)	
	Very much	43(16.54)	17(6.58)	
4.	Do you feel that your hospital staff have sufficient knowledge to follow the COVID-19 appropriate behaviour?			
	Not at all	48(18.46)	51(19.76)	12.22(.0066)*
	Normal	128(49.23)	152(58.91)	
	Much	61(23.46)	48(18.60)	
	Very much	23(8.85)	7(2.71)	
6.	Are you anxious to be infected with COVID-19 because of your profession?			
	Not at all	24(9.23)	18(6.97)	1.97(.578)
	Normal	82(31.54)	73(28.29)	
	Much	77(29.62)	81(31.39)	
	Very much	77(29.62)	86(33.33)	
7.	Are you anxious about the shut-down of your privately owned clinic or loosening your job because of the COVID-19 outbreak?			
	Not at all	56(21.54)	54(20.93)	.820(.844)
	Normal	81(31.15)	73(28.29)	
	Much	63(24.23)	70(27.13)	
	Very much	60(23.08)	61(23.64)	
8.	Are you anxious about your career as a dentist because of COVID-19 outbreak?			
	Not at all	60(23.08)	43(16.66)	9.26(.026)*
	Normal	73(28.08)	62(24.03)	
	Much	65(25.00)	62(24.03)	
	Very much	62(23.85)	91(35.27)	

The overall general coping skills and self-efficacy of the dentists were adequate during the COVID-19 pandemic (Table 5).

**Table 5:** General Self-efficacy Scale (n=518) **P<0.05, **P<0.01, ***P<0.001

S. No	Questions	Not At All True n(%)	Hardly True n(%)	Moderately True n(%)	Exactly True n(%)	Chi-square test/t-test (p)
1.	I can always manage to solve difficult problems if I try hard enough.	11(2.12)	39(7.53)	268(51.74)	200(38.61)	
	Postgraduate specialities	7(63.64)	19(48.72)	180(67.16)	146(73.00)	9.13(.027)*
	Undergraduate	4(36.36)	20(51.28)	88(32.84)	54(27.00)	
2.	If someone opposes me, I can find the means and ways to get what I want.	28(5.41)	75(14.48)	301(58.11)	114(22.01)	
	Postgraduate specialities	19(67.86)	52(69.33)	209(69.44)	72(63.16)	1.57(.665)
	Undergraduate	9(32.14)	23(30.67)	92(30.66)	42(36.84)	
3.	It is easy for me to stick to my aims and accomplish my goals.	13(2.51)	59(11.39)	303(58.49)	143(27.61)	
	Postgraduate specialities	7(53.85)	34(57.63)	218(71.95)	93(65.03)	6.855(.076)
	Undergraduate	6(46.15)	25(42.37)	85(28.05)	50(34.93)	
4.	I am confident that I could deal efficiently with unexpected events.	16(3.09)	46(8.88)	316(61.00)	140(27.03)	
	Postgraduate specialities	9(56.25)	25(54.35)	227(71.84)	91(65.00)	7.66(.053)
	Undergraduate	7(43.75)	21(45.65)	89(28.16)	49(35.00)	
5.	Thanks to my resourcefulness, I know how to handle unforeseen situations.	16(3.09)	74(14.29)	303(58.49)	125(24.13)	
	Postgraduate specialities	11(68.75)	51(68.92)	209(68.98)	81(64.80)	.752(.860)
	Undergraduate	5(31.25)	23(31.08)	94(31.02)	44(35.20)	
6.	I can solve most problems if I invest the necessary effort.	6(1.16)	40(7.72)	241(46.53)	231(44.59)	
	Postgraduate specialities	3 (50)	23 (57.5)	164 (68.05)	162 (70.12)	3.39(.334)
	Undergraduate	3 (50)	17 (42.5)	77 ( 31.95)	69 (29.88)	
7.	I can remain calm when facing difficulties because I can rely on my coping abilities.	23(4.44)	61(11.78)	280(54.05)	154(29.73)	
	Postgraduate specialities	13(56.52)	35(57.38)	199(71.07)	105(68.18)	5.767(.1234)
	Undergraduate	10(43.48)	26(42.62)	81(28.93)	49(31.82)	
8.	When I am confronted with a problem, I can usually find several solutions.	9(1.74)	71(13.71)	307(59.27)	131(25.29)	
	Postgraduate specialities	6(66.67)	39(54.93)	217(70.68)	90(68.70)	6.622(.0849)
	Undergraduate	3(33.33)	32(45.07)	90(29.32)	41(31.30)	
9.	If I am in trouble, I can usually think of a solution.	7(1.35)	34(6.56)	288(55.60)	189(36.49)	
	Postgraduate specialities	5(71.43)	20(58.82)	208(72.22)	119(62.96)	5.9117(.1159)
	Undergraduate	2(28.57)	14(41.18)	80(27.78)	70(37.04)	
10.	I can usually handle whatever comes my way.	7(1.35)	54(10.42)	291(56.18)	166(32.05)	
	Postgraduate specialities	5(71.43)	31(57.41)	204(70.10)	112(67.47)	3.432(.329)
	Undergraduate	2(28.57)	23(42.59)	87(29.90)	54(32.53)	

## Discussion

The present cross-sectional study assessed the level of psychological distress, anxiety, and fear of getting infected amongst dental practitioners, while working during the COVID 19 pandemic outbreak in India, and assessed the possible factors associated with it. For this purpose, a questionnaire composed of closed-ended questions was used to gather information about psychological distress, anxiety, and fear of getting infected among dental practitioners while working during the COVID 19 pandemic. The findings confirmed our hypothesis, suggesting that dental practitioners exhibited an elevated risk of developing psychological distress. Similar studies involving dental staff in countries like Israel [13], India [6], Italy [14], Poland [15], and Saudi Arabia [16] also report confirming higher psychological distress rates due to the COVID-19 pandemic.

Since it has been established that the primary route for transmission of coronavirus is through the airborne spread via aerosols formed during dental procedures [17], other possible routes are contact spread, and contaminated surfaces spread [18]. This enhances the likelihood of dental practitioners getting infected and further spreading the virus. The present study found that many dental practitioners feared getting infected by their patients or co-workers. The rapid spread of the coronavirus terrified the rest of the population as they were at risk of getting infected by other individuals in the community [4]. The majority of dental practitioners were fearful of providing treatment to any symptomatic patient. Since the coronavirus rapidly infected such a large number of individuals in a short time in almost every country, the fear of getting infected by a patient has supporting empirical evidence.

In the present study, 61.97% of the dental practitioners expressed much concern about getting COVID-19 infection during practice. In comparison with other countries, it was around 85% of 356 dental practitioners and 71.9% of 1237 participants in Italy [14,19] and Norway [20], respectively. Also, in the present study, around 50% of the participants expressed concern about losing their job or closure of practice due to the COVID-19 pandemic. In contrast, it was comparatively higher, i.e. 67.02%, 89.6%, and 80% in studies from India, Italian, and America [5,14,19,20], where dentists reported more concerns about professional future, practice closure, and economic issues. The possible reasons were earning from other sources or support from other family members both psychologically and economically in our study group. The current study found that females had higher career-related anxiety compared to their male counterparts.

Many dental practitioners in India wanted to shut down their privately owned clinics which may have had significant economic implications and may have reflected in the level of anxiety. During the COVID 19 pandemic, patients suffering from dental pain experienced delays in dental care due to a multi-visit treatment plan and teleconsultations.

The current guidelines on the COVID-19 outbreak have recommended deferring all non-essential and elective dental treatment until the situation is regressing or under control. Only emergency dental treatments have to be done, such as for patients suffering from severe pain, swelling, bleeding, and trauma [7]. Another genuine fear that dentists have was of carrying infections from their dental practices to their families. The prolonged incubation period during the asymptomatic phase and the longevity of coronavirus on various surfaces from a few hours to a few days make it particularly difficult to limit its transmission [18]. The anxiety and fear of getting quarantined due to coronavirus infection also legitimise nervousness. The burden on the healthcare system and the cost incurred during treatment also put one's mind under stress.

In our study, it was found that self-efficacy was adequate and comparable in both postgraduate and undergraduate dental practitioners (Table 5).

Limitations, being an online survey, posed several limits to sample coverage. The study was subject to selection bias and sampling error, as participants were approached using social media, dedicated mailing lists, and forums. Capturing a relatively younger population, perhaps due to the accessibility of smartphones and the internet, affected the sample. Missing out older practitioners, who may be more vulnerable to COVID-19-related stress, could have caused under-reporting psychological distress. The questionnaire used to assess attitude and knowledge was not adequately validated and just validated by a pilot survey among 10 doctors of the same institute.

Strengths of this study include a large sample size; responses were conducted during the peak of the first wave of COVID-19 immediately after a lockdown in India to minimise recall biases and enable the assessment of the actual situation.

## Conclusions

It can be concluded that dental practitioners suffered considerable impact due to the COVID-19 pandemic. Although there was a variation in the stressors and psychosocial factors, dental practitioners suffered significant career-related anxiety. Surprisingly, postgraduate dental practitioners continued their dental practice with their clinical work by performing non-aerosol generating procedures despite significant COVID-19-related anxiety, perhaps due to better self-efficacy skills. Hence, apart from COVID-19 safety guidelines and policies, there is a need to enhance coping and self-efficacy strategy among dental practitioners.
